# Incidence and mortality of bone cancer among children, adolescents and young adults of Brazil

**DOI:** 10.6061/clinics/2019/e858

**Published:** 2019-05-27

**Authors:** Nathalie Vieira Balmant, Rejane de Souza Reis, Marceli de Oliveira Santos, Mariana Maschietto, Beatriz de Camargo

**Affiliations:** I Instituto Nacional do Cancer, Rio de Janeiro, RJ, BR.; II Fundacao do Cancer, Rio de Janeiro, RJ, BR.; III Coordenacao de Prevencao e Vigilancia, Instituto Nacional do Cancer, Rio de Janeiro, RJ, BR.; IV Laboratorio Nacional de Biociencias, Centro Nacional de Pesquisa em Energia e Materiais, Campinas, SP, BR.; V Centro de Pesquisa, Instituto Nacional de Cancer, Rio de Janeiro, RJ, BR.

**Keywords:** Brazil, Incidence, Mortality, Bone Cancer, Children, Adolescents, Young Adults

## Abstract

**OBJECTIVES:**

Bone cancers occur frequently in children, adolescents, and young adults aging 15 to 29 years. Osteosarcoma and Ewing sarcoma are the most frequent subtypes in this population. The aim of this study was to describe incidence and mortality trends of bone cancers among Brazilian children, adolescents and young adults.

**METHODS:**

Incidence information was obtained from 23 population-based cancer registries. Mortality data were extracted from the Atlas of Cancer Mortality from 1979 to 2013. Specific and adjusted rates per million were analyzed according to gender, morphology and age at diagnosis. Median rates were used as a measure of central tendency. Joinpoint regression was applied to analyze trends.

**RESULTS:**

Median incidence rates were 5.74 and 11.25 cases per million in children and young adults respectively. Osteosarcoma in the 15-19 years aged group had the highest incidence rates. Stable incidence rates were observed among five registries in 0-14 year's age group. Four registries had a decreased incidence trend among adolescents and young adults. Median mortality rates were 1.22 and 5.07 deaths per million in children and young adults respectively. Increased mortality was observed on the North and Northeast regions. Decreased mortality trends were seen in the South (children) and Southeast (adolescents and young adults).

**CONCLUSION:**

Osteosarcoma and Ewing Sarcoma are the most incident bone cancers in all Brazilian regions. Bone cancers showed incidence and mortality patterns variation within the geographic regions and across age groups, although not significant. Despite limitations, it is crucial to monitor cancer epidemiology trends across geographic Brazilian regions.

## INTRODUCTION

Bone cancers derive from primitive mesenchymal cells comprising a variety of tumors including osteosarcoma (OS), Ewing sarcoma (ES), chondrosarcoma (CS) and other rarer subtypes. Bone cancers account for 3 to 8% of malignancies affecting the 0 to 19 year age group and for 3% of all cancer cases among adolescents and young adults (AYA) aged 15 to 29 years (1,2).

OS and ES are the most frequent morphological subtypes of bone cancers presenting a worldwide distribution of about 20 to 40% and 6 to 8% of all bone tumors, respectively (3,4). Except in the first decade of life, OS occurs more frequently in males. Approximately 80% of patients with ES are younger than 20 years, with the peak incidence occurring during the second decade of life. CS is the most common subtype in older patients (3,5).

Despite improvements in treatment, children and adolescents with bone cancers still present a high mortality, being the third most frequent cause of death related to cancer in England (6). In Brazil, mortality in 15-19 years age group due to bone cancers was reported at five cases per million, decreasing to three cases per million in 20-29 years age group (7).

In this study, our aim was to describe incidence and mortality trends of bone cancers, considering the subtypes, among children (0-14 years) and AYA (15-29 years) in Brazil.

## METHODS

Information about incidence was obtained from 23 population-based cancer registries (PBCR) covering the five geographic regions of Brazil: North (four PBCR), Northeast (seven PBCR), Midwest (three PBCR), Southeast (six PBCR) and South (three PBCR) that correspond to 23% of the total Brazilian population (North, 32%; Northeast, 22%; Midwest, 39%; Southeast, 22% and South 13%). Data quality indicators can be seen in Supplemental Table S1.

Morphological subtypes of bone cancers as well as sites of involvement were classified according to the third edition of International Classification of Childhood Cancer (ICCC-3) among children, and according to the WHO Classification for AYA (8,9).

To evaluate the frequency of bone cancers in different topographies, all cases were classified according to the codes of the International Classification of Diseases for Oncology – third edition (ICD-O-3): C400 – long bones of the upper limb, scapula and associated joints; C401 – short bones of the upper limbs and associated joints; C402 – long bones of the lower limbs and associated joints; C403 – short bones of the lower limbs and associated joints; C408 – overlapping lesions of bones, joints and articular cartilage of the limbs; C409 – bone of the limbs, not otherwise specified (NOS); C410 – bones of the skull and face and associated joints; C411 – mandible; C412 – vertebral column; C413 – rib, sternum, clavicle and associated joints; C414 – pelvic bones, sacrum, coccyx and associated joints; C418 – overlapping lesions of bones, joints and articular cartilage; and C419 – bone, NOS.

Data for the mortality analysis were extracted from the On-line Atlas of Cancer Mortality databases (https://mortalidade.inca.gov.br) from 1979 to 2013, as provided by the Brazilian Health Mortality Information System (SIM). The cause of death was based on the International Classification of Diseases and Related Health Problems versions 9 (ICD-9; 1979–95) and 10 (ICD-10; 1996–2008). The codes for bone cancers were C-40 (bone and joints of the limbs) and C-41 (bones and joints of other sites). Mortality considered the five geographic regions of Brazil.

Incidence trends were evaluated in six PBCR for children and in 12 PBCR for AYA which had eight years of database consolidation. PBCR with rate values of zero were excluded. Mortality trends were analyzed according to the five geographic regions of Brazil.

### Statistical analyses

Age-specific incidence rates (ASIR) per million population were analyzed for each morphological subtype of bone tumor according to gender and age at diagnosis (stratified into: 0 to 4 years, 5 to 9 years, 10 to 14 years, 15 to 19 years, 20 to 24 years, and 25 to 29 years). Age-adjusted incidence rates (AAIR) and age-adjusted mortality rates (AAMRs) were performed in those aged 0 to 14 years and 15 to 29 years and estimated based on the world population proposed for groups aged less than 30 years (10). Median incidence rates were used to measure central tendency and to obtain an overall assessment of incidence rates. Age-specific mortality rates (ASMRs) were calculated according to deaths by bone cancers.

To identify significant changes in incidence and mortality trends, Joinpoint regression analysis was performed, and the annual average changes (Average Annual Percent Change [AAPC]) were estimated. The best cut-point period for measuring the trends is described elsewhere. Significance was determined with the Monte Carlo permutation method.

## RESULTS

### Distribution by site and subgroups (ICCC-3/ WHO) of bone tumors

According to morphological subtypes, long bones of the lower limbs was the most common affected site, corresponding to 66% OS and 24% ES, followed by long bones of the upper limbs (10% OS and 11.2% SE) and pelvic bones (3% OS and 17% ES).

### Incidence rates

The AAIR in children was 5.74 cases per million, with highest rates observed in Porto Alegre, Distrito Federal and Curitiba. The 10-14 year age group had the highest ASIR. Among the AYA group, the median AAIR was 11.25 cases per million. The 15-19 year age group had the highest rates, decreasing among those aged 20-29 years. The highest incidence rates among the AYA group were seen in Florianopolis, Joao Pessoa, and Goiania (Table 1).

**Table 1:** Incidence rates per million (ASIR; AAIR) of bone tumors in children, adolescents, and young adults according to age group and region.

Region	PBCR	0-4 years		5-9 years		10-14 years		0-14 years			15-19 years		20-24 years		25-29 years		15-29 years		
		n	ASIR	n	ASIR	n	ASIR	n	AAIR	CI (95%)	n	ASIR	n	ASIR	n	ASIR	n	AAIR	CI (95%)
North	Belem (2005-2009)	*	1.71	*	6.12	13	19.44	18	8.24	(4.41; 12.07)	13	17.70	6	8.48	5	8.26	24	11.79	(7.06; 16.52)
	Manaus (2002-2006)	*	1.06	*	2.29	6	7.30	9	3.24	(1.12; 5.37)	19	23.16	7	8.77	7	10.50	33	14.63	(9.62; 19.63)
	Palmas (2008-2012)	*	0.00	*	0.00	*	19.56	*	5.64	(-2.69; 8.28)	*	9.69	*	0.00	*	27.86	*	12.07	(0.18; 23.96)
	Roraima (2006-2010)	*	0.00	*	0.00	*	4.26	*	1.23	(-1.19; 3.65)	*	5.07	*	11.55	*	6.63	*	7.32	(0.13; 14.51)
Northeast	Aracaju (2007-2011)	*	4.73	*	0.00	*	8.79	*	4.38	(-0.65; 9.41)	*	11.65	*	3.73	5	20.44	9	11.78	(4.07; 19.48)
	Fortaleza (2002-2006)	*	1.81	5	4.49	21	17.75	28	7.37	(4.61; 10.12)	15	12.72	10	9.30	8	8.62	33	10.35	(6.81; 13.89)
	Joao Pessoa (2006-2010)	*	3.82	*	3.56	*	9.90	5	5.37	(0.58; 10.17)	8	24.74	*	9.53	5	17.99	16	17.54	(8.93; 26.14)
	Natal (2001-2005)	*	0.00	7	20.50	*	7.93	10	9.00	(3.39; 14.61)	*	10.15	*	5.60	*	0.00	6	5.42	(1.08; 9.77)
	Recife (2006-2010)	*	5.50	6	10.07	6	9.38	15	8.06	(3.92; 12.19)	8	11.30	6	8.60	6	9.32	20	9.84	(5.52; 14.15)
	Salvador (2001-2005)	*	0.91	*	3.69	14	11.92	19	4.92	(2.68; 7.17)	19	13.48	9	6.53	8	7.03	36	9.06	(6.09; 12.03)
	Teresina (2002-2006)	*	0.00	*	0.00	*	6.94	*	1.96	(-0.26; 4.19)	10	5.79	8	5.80	*	1.92	20	4.56	(2.54; 6.58)
Midwest	Cuiaba (2003-2007)	*	0.00	*	0.00	8	29.66	8	8.56	(2.62; 14.49)	5	18.22	*	0.00	*	9.14	7	9.36	(2.42; 16.30)
	Distrito Federal (1998-2002)	*	7.37	5	6.43	15	18.84	26	10.50	(6.44; 14.56)	15	16.86	11	12.20	6	7.61	33	13.1	(8.62; 17.59)
	Goiania (2005-2009)	*	0.00	*	4.03	9	17.40	11	6.31	(2.57; 10.05)	13	22.03	6	9.82	7	13.55	26	15.59	(9.56; 21.63)
Southeast	Belo Horizonte (2004-2008)	*	4.34	7	7.64	16	16.32	27	8.90	(5.51; 12.29)	19	17.12	20	17.25	*	3.03	42	14.69	(10.24; 19.14)
	Campinas (2001-2005)	*	0.00	*	7.63	*	4.68	5	3.80	(0.46; 7.15)	6	13.27	*	6.35	*	4.65	11	8.37	(3.40; 13.34)
	Grande Vitoria (2008-2012)	*	7.67	*	0.00	7	10.01	12	5.74	(2.44; 9.03)	9	11.83	5	6.96	*	4.96	17	8.2	(4.29; 12.11)
	Jahu (2009-2013)	*	0.00	*	0.00	*	0.00	*	0.00	–	*	16.93	*	15.32	*	0.00	*	11.25	(-4.50; 27.00)
	Poços de Caldas (2007-2011)	*	0.00	*	0.00	*	0.00	*	0.00	–	*	15.93	*	0.00	*	0.00	*	5.96	(-5.72; 17.65)
	Sao Paulo (2006-2010)	*	0.98	32	7.46	66	15.33	102	7.24	(5.83; 8.65)	75	4.20	49	2.76	45	2.71	169	3.26	(2.77; 3.75)
South	Curitiba (2006-2010)	5	8.31	5	7.42	9	12.60	19	9.37	(5.06; 13.68)	15	19.91	6	7.56	6	8.10	27	13.35	(8.31; 18.40)
	Florianopolis (2008-2012)	*	0.00	*	0.00	*	13.64	*	3.93	(-1.52; 9.39)	*	23.89	*	20.63	*	5.20	9	19.63	(6.78; 32.47)
	Porto Alegre (2002-2006)	*	5.56	*	5.66	15	26.97	21	11.77	(6.69; 16.84)	12	19.18	9	14.54	*	5.65	24	13.21	(7.91; 18.51)
MEDIAN			0.98		4.03		11.92		5.74			15.93		8.48		7.03		11.25	

* Absolute value <5 cases.

AAIR: Age-adjusted incidence rates; ASIR: Age-specific incidence rates.

**Figure 1:**
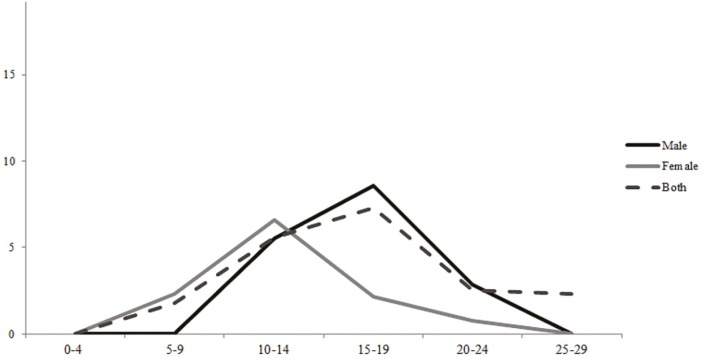
Age-specific incidence rates (ASIR) of Osteosarcoma in children, adolescents and young adults in Brazil, according to gender and age group.

According to gender, median incidence rates of all bone cancers were similar for children (AAIR: 5.02 per million in males; 5.85 per million in females). For AYA, a higher incidence rate was seen for males (AAIR: 12.24 per million in males; 7.54 per million in females). Regarding the distribution of bone tumor morphological subtypes, OS was more frequent (47.3%), followed by ES (24.5%), other specific/non-specific tumors (21.8%) and CS (6.4%). Among other specific/non-specific tumors, more than 90% corresponded to non-specific bone cancers for both age groups. The incidence analysis by morphological subtypes of bone cancers can be seen in Table 2. Median incidence rates of OS among children was higher in females (AAIR: 2.14 per million in males; 3.12 per million in females) while males had the highest incidence rates among AYA (AAIR: 4.69 per million in males; 2.36 per million in females). ES showed a slight difference between genders in both children (AAIR: 1.11 per million in males; 1.62 per million in females) and AYA (AAIR: 1.81 per million in males; 1.76 per million in females). Incidence rates of CS had no differences between genders. The incidence peak of OS in females was earlier (10 to 14 years) compared to males (15 to 19 years) (Figure 1).

**Table 2:** Distribution and age-ajusted incidence rates (AAIR) of morphological subtypes of bone tumors in children, adolescents and young adults, according to age group and region.

Region	PBCR	Osteosarcoma				Chondrosarcoma				Ewing Sarcoma				Other specific and non-specific			
		0-14		15-29		0-14		15-29		0-14		15-29		0-14		15-29	
		(%)	AAIR	(%)	AAIR	(%)	AAIR	(%)	AAIR	(%)	AAIR	(%)	AAIR	(%)	AAIR	(%)	AAIR
North	Belem (2005-2009)	50.0	4.01	58.3	6.88	0.0	0.00	8.3	0.96	44.4	3.78	12.5	1.48	5.6	0.45	20.8	2.46
	Manaus (2002-2006)	66.7	2.12	42.4	6.24	0.0	0.00	3.0	0.42	22.2	0.71	24.2	3.64	11.1	0.41	30.3	4.33
	Palmas (2008-2012)	50.0	2.8	0.0	0.00	0.0	0.00	0.0	0.00	50.0	2.84	50.0	6.33	0.0	0.00	50.0	5.74
	Roraima (2006-2010)	0.0	0.00	25.0	1.78	0.0	0.00	0.0	0.00	0.0	0.00	0.0	0.00	100.0	1.23	75.0	5.54
Northeast	Aracaju (2007-2011)	33.3	1.29	55.6	6.75	0.0	0.00	0.0	0.00	66.7	3.09	33.3	3.78	0.0	0.00	11.1	1.25
	Fortaleza (2002-2006)	75.0	5.35	72.7	7.45	0.0	0.00	3.0	0.3	17.9	1.39	15.2	1.62	7.1	0.62	9.1	0.99
	Joao Pessoa (2006-2010)	80.0	3.93	18.8	3.48	0.0	0.00	37.5	6.46	0.0	0.00	6.3	1.09	20.0	1.44	37.5	6.51
	Natal (2001-2005)	60.0	5.25	33.3	1.86	0.0	0.00	16.7	0.93	20.0	1.83	33.3	1.77	20.0	1.91	16.7	0.86
	Recife (2006-2010)	40.0	3.12	35.0	3.42	6.7	0.44	20.0	1.93	26.7	2.27	10.0	0.98	26.7	1.11	35.0	3.51
	Salvador (2001-2005)	63.2	3.01	69.4	6.25	10.5	0.50	13.9	1.29	21.1	1.19	13.9	1.25	5.3	0.22	2.8	0.27
	Teresina (2002-2006)	33.3	0.64	55.0	2.41	0.0	0.00	0.0	0.00	33.3	0.67	15.0	0.73	33.3	0.66	30.0	1.43
Midwest	Cuiaba (2003-2007)	50.0	4.26	42.9	4.06	37.5	3.19	0.0	0.00	0.0	0.00	28.6	2.7	12.5	1.11	28.6	1.6
	Distrito Federal (1998-2002)	19.2	1.9	21.2	2.78	11.5	1.06	18.2	2.57	3.8	0.38	9.1	1.11	65.4	3.58	51.5	6.65
	Goiania (2005-2009)	72.7	4.54	61.5	9.86	0.0	0.00	3.9	0.68	27.3	1.77	26.9	3.88	0.0	0.00	7.7	1.17
Southeast	Belo Horizonte (2004-2008)	48.1	3.89	40.5	6.04	0.0	0.00	9.5	1.41	29.6	2.98	19.0	2.77	22.2	2.03	31.0	4.47
	Campinas (2001-2005)	40.0	1.46	45.5	3.69	0.0	0.00	18.2	1.48	60.0	2.34	9.1	0.86	0.0	0,00	27.3	2.34
	Grande Vitoria (2008-2012)	33.3	1.87	47.1	3.95	0.0	0.00	0.0	0.00	41.7	2.22	29.4	2.41	25.0	1.65	23.5	1.4
	Jahu (2009-2013)	0.0	0.00	50.0	4.81	0.0	0.00	0.0	0.00	0.0	0.00	50.0	6.44	0.0	0.00	0.0	0.00
	Poços de Caldas (2007-2011)	0.0	0.00	0.0	0.00	0.0	0.00	0.0	0.00	0.0	0.00	0.0	0.00	0.0	0.00	100.0	5.96
	Sao Paulo (2006-2010)	53.9	3.83	42.0	1.38	0.0	0.00	7.7	0.24	24.5	1.8	29.6	0.67	21.6	0.8	20.7	0.96
South	Curitiba (2006-2010)	31.6	2.61	40.7	5.43	0.0	0.00	3.7	0.46	52.6	5.02	44.4	6.01	15.8	1.75	11.1	1.45
	Florianopolis (2008-2012)	50.0	3.93	33.3	6.72	0.0	0.00	11.1	2.07	0.0	0.00	44.4	8.78	50.0	0.00	11.1	2.07
	Porto Alegre (2002-2006)	38.1	4.39	58.3	7.91	0.0	0.00	8.3	1.11	47.6	5.59	16.7	2.1	14.3	1.79	16.7	2.09
MEDIAN			3.01		4.06		0.00		0.46		1.77		1.77		0.66		2.07

* Absolut value <5 cases.

AAIR: Age-adjusted incidence rates.

### Mortality rates

ASMR was higher among the group aged 15-19 years. The Midwest region had the highest ASMR in most age groups. AAMR was higher in AYA compared to children group (Table 3).

**Table 3:** Age-specific and age-adjusted mortality rates of bone tumors in children, adolescents and young adults from Brazil, SIM 2009-2013.

Brazilian	0-4	5-9	10-14	15-19	20-24	25-29	0-14	15-29
Region	ASMR	ASMR	ASMR	ASMR	ASMR	ASMR	AAMR	AAMR
North	0.76	1.31	3.75	6.27	4.82	2.67	1.16	4.65
Northeast	0.50	1.63	3.93	7.00	4.51	2.87	1.21	4.88
Midwest	0.91	2.93	5.24	10.01	5.30	5.07	1.65	6.92
Southeast	0.86	1.63	3.44	6.64	4.40	2.86	1.09	4.72
South	0.56	1.60	4.78	8.45	5.66	2.78	1.46	5.74
BRAZIL	**0.70**	**1.69**	**3.93**	**7.22**	**4.71**	**3.01**	**1.22**	**5.07**

ASMR: Age-specific mortality rates; AAMR: Age-adjusted mortality rates.

### Incidence and mortality trends

**Figure 2:**
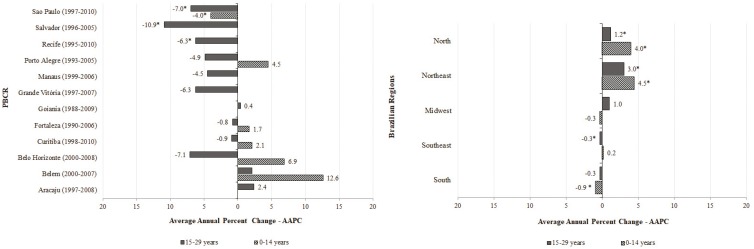
Joinpoint regression analysis of incidence and mortality trends by bone tumors in children, adolescents and young adults (0-29 years) in Brazil, according to PBCR and Brazilian regions.

Among children, five PBCR showed an increased trend in incidence, although it was not significant. Only one PBCR (São Paulo) showed a significant decreased trend in incidence (AAPC: −4.0%). Among the AYA group, three PBCR had a significant decrease in trends (Recife, −6.3%; Salvador, −10.9%; and São Paulo, −7.0%) while most PBCR only showed a decreased non-significant trend in incidence. Mortality due to bone cancers among children had an increased trend in the North and Northeast regions (AAPC: 4.0% and 4.5%, respectively). A significant decreased trend in mortality (AAPC: −0.9%) was seen in the South region. The North and Northeast regions also demonstrated a significant increased trend in mortality (AAPC: 1.2% and 3.0%, respectively) and the Southeast showed a stable pattern (AAPC: −0.3%) among the AYA group (Figure 2).

## DISCUSSION

This study summarized the incidence and mortality rates of bone cancers in Brazil. We observed that while there was less variation in bone cancer subtypes, with OS and ES being prevalent in all regions and over time, there was incidence and mortality patterns variation within the geographic regions and across age groups, although not always significant.

Data presented here were collected from PBCR, allowing for the study of cancer trends and capturing cases from the five geographic regions across the country. However, on top of not covering all cases within Brazil, the 23 PBCR may be limited due to incomplete registration, preferential report of malignant diseases, diagnosis (histology) confirmation, region extent and different periods of coverage. Nevertheless, the most up-to-data were carefully examined and we believe that data presented here are a true representation, although not exact, of the bone cancer incidence and mortality in Brazil.

The incidence rates of bone cancers affecting children and AYA are similar and remain stable across countries. A recent publication from the International Agency for Research on Cancer, that included five PBCR from Brazil, reported age-adjusted incidence rates of bone cancer in South America (5.80 per million in ages 0-14 years and 16.50 per million in ages 15-19 years), similar to our data (5,11). Previously, Brazil had median incidence rates for all bone cancers in children and adolescents (0-19 years) of 9.53 cases per million according to 14 PBCR and 9.38 cases per million in AYA according to 21 PBCR (12,13). In summary, Brazilian median incidence rates for all bone cancers followed the international pattern, where the highest rates were seen between the ages of 10 and 19 years then decreasing gradually after 20 years of age.

Considering only the current study, children showed a stable incidence rate, except for Sao Paulo that reported a decreased incidence rate. Historically, Sao Paulo, as the biggest Brazilian metropolitan city, receives patients from all over the country because has more specialized cancer centers. With the implementation of specialized centers capable of treating children with bone cancers across the country, less people move from their homes to receive treatment. Moreover, the Brazilian Osteosarcoma Treatment Group (BOTG) and the Brazilian Collaborative Study Group for the Treatment of Ewing Sarcoma Family Tumors (EWING1) have been collaborating to improve diagnosis and treatment (14,15). Considering the size of Brazilian population, for children and AYA, we would expect 360 and 180 new cases of OS and ES, respectively, per year. During 15 years of experience from BOTG, 778 cases (51 cases per year) were registered, corresponding to 14% of the estimated number of cases (25 participating institutions from 15 cities). During the past eight years, 175 cases were included in the EWING1 register corresponding to 12% of estimated number of cases (15 institutions from Brazil and one institution from Uruguay). The efforts from the cooperative groups resulted in an increasing number of registered cases over the years, despite still being far below the expected number per year (15,16).

Among 25 countries distributed over five continents (Brazil not included), the median incidence rate of OS was 4.20 cases per million in males and 3.10 cases per million in females aged 0 to 24 years (4). Among the AYA group, incidence rates of OS were also similar for different countries (11,17). However, we cannot make a full evaluation of the incidence rates by morphological subtypes over larger periods because these have not been described in Brazil since 1993, when an incidence rate of OS (3.9 per million) and ES (2.5 per million) among ages 0-14 years was described for Sao Paulo (18).

In agreement with previous findings, OS and ES present different skeletal site distributions, with OS occurring more commonly in the long bones of lower limbs while ES were diagnosed more commonly in the long bones of upper limbs. Accordingly, these sites are adjacent to the growth plate, characterized by rapid growth during the adolescent spurt, reinforcing the relationship between the high cell cycle turnover due to bone growth and OS development (3,19). According to this hypothesis, we observed higher incidence rate for bone cancers among boys from 15-19 years old in the South and Midwest region in Brazil where the median height is higher (www.sidra.ibge.gov.br/tabela/2645).

Regarding the other bone cancers subtypes, we observed a higher incidence rate of NOS compared to the literature. Diagnosis of bone cancers is usually performed using a combination of morphological and radiological features. However due to their rarity, only a centralized pathology and radiology reviews, secondary to the local review, would improve accuracy in diagnosis (20). Moreover, with the advance of basic and translational research, several biomarkers are being explored to meliorate the diagnosis (21).

Apart from a few established risk factors for OS and ES, little is known about the etiological pathways for bone cancers and their subtypes (3). Few cases of OS are attributed to germline mutations. Mutations in *TP53* (Li-Fraumeni syndrome) and *RB1* (familial retinoblastoma syndrome) increase the risk to develop OS in 15- and 500-1000-fold compared with general population (17). Varying patterns of incidence of bone cancers are observed across countries, with a peak in teenage years observed particularly in high and mid-range incidence countries, suggesting some influence of the environment through epigenetic mechanisms (22).

We observed a shift in the incidence with increasing and decreasing trend incidences of bone cancers in children and AYA groups, respectively. Although we have to be careful with this observation because most data did not show statistical significance, we might consider that age of puberty may be falling (23). An increasing trend of OS incidence was observed for females at younger ages, raising the question whether the earlier exposure to estrogen (due to the earlier puberty) could be involved with this shift. For instance, in older women from USA and UK, an increasing incidence rates of CS was reported for females but unchanged for males, coincident with the introduction of exogenous estrogen exposure by oral contraceptives and hormonal therapy when they were younger, giving some evidence that the relationship between estrogen exposure may have a role in bone cancer development (19).

There is a lack of information about mortality trends in bone cancers in the literature, usually related to the availability of high-quality vital statistics (24). Mortality rates are affected by the quality of data on diagnosis and death certification, socioeconomic characteristics, and medical facilities specialized in pediatric oncology (25). The Mortality Information System was created in 1975 in Brazil and in 2005, the Ministry of Health included a wide range of actions to improve the quality of death certifications, mainly in the North and Northeast regions. This improvement coincided with a decrease of ill-defined causes of mortality in 2010 (7,25). Agreeing with this technical bias, data from the BOTG comparing overall survival among children (aged less than 12 years) and AYA does not appear to be significantly different, suggesting that the participation in studies could improve prognosis (26).

Mortality rates due to bone cancers increased with the advanced age. Among the children, AAMR was 1.22 cases per million compared to 5.07 cases per million in AYA, with no differences related to gender. A previous report performed in eight countries (including Brazil) showed an AAMR (2005-2007) of 1.20 cases per million in males and 3.58 cases per million in females in children aged 0 to 14 years, with Argentina showing the highest rates (2.10 and 2.40 cases per million in males and females, respectively) (24).

Mortality rates due to bone cancers in the 15-29 years age group in USA were five cases per million in males and 2.50 cases per million in females. Mortality trends in the 0-14 year age group presented a slight increase (AAPC=0.3%) (15,16). In our analysis, the increase in AAPC was higher mainly in the North and Northeast regions in both age groups, as previously reported (7).

In Great Britain, a greater risk of dying early for OS and ES was associated to living in more rural areas, which was linked to lower socio-economic factors or distance from specialist treatment centers (27). The most important prognostic factor for overall survival is the presence of metastases at diagnosis (19). BOTG and EWING1 demonstrated that the frequency of metastatic disease in Brazilians is high compared to other populations.

We discussed some limitations of this study throughout the text. It is worth to note the limitations of the 23 PBCR regarding the Brazilian population and territory size. Also, due to the rarity of bone cancers, especially subtypes other than OS, ES and CS a curate diagnosis remains challenging. Moreover, this rarity also prevented us to proper explore probable environmental factors potentially associated with bone cancer incidence rates across Brazilian regions. However, it is crucial to monitor cancer incidence and mortality trends across geographic regions, even if data are not complete, as this effort will enable to generate hypothesis related to genetic, epigenetic and environmental risk factors and point to at high risk groups that may need closer monitoring.

## Appendix

**Supplemental Table S1:** Data quality indicators of 23 Brazilian PBCR and your respective periods of information, according to International Agency for Research in Cancer (IARC).

PBCR	0-19 years		15-29 years	
	MV%	DCO%	MV%	DCO%
Aracaju (2007-2011)	100.0	0.0	100.0	0.0
Belém (2005-2009)	87.9	6.1	84.0	12.0
Belo Horizonte (2003-2007)	91.3	6.5	82.0	7.7
Campinas (2001-2005)	100.0	0.0	72.7	27.3
Cuiabá (2003-2007)	100.0	0.0	100.0	0.0
Curitiba (2006-2010)	97.14	2.86	96.6	3.4
Distrito Federal (1998-2002)	76.2	7.1	63.6	15.2
Florianópolis (2008-2012)	100.0	0.0	88.9	0.0
Fortaleza (2002-2006)	86.62	10.84	97.1	2.9
Goiânia (2005-2009)	–	–	–	–
Grande Vitória (2008-2012)	100.0	0.0	81.3	18.7
Jahu (2009-2013)	100.0	0.0	100.0	0.0
João Pessoa (2006-2010)	100.0	0.0	100.0	0.0
Manaus (2002-2006)	96.55	3.45	87.9	6.1
Natal (2001-2005)	78.57	7.14	83.3	0.0
Palmas (2008-2012)	100.0	0.0	75.0	25.0
Poços de Caldas (2007-2011)	–	–	50.0	0.0
Porto Alegre (2002-2006)	84.85	6.06	76.0	16.0
Recife (2006-2010)	95.0	5.0	81.3	6,3
Roraima (2006-2010)	100.0	0.0	40.0	20.0
Salvador (2001-2005)	84.62	5.13	76.9	2.6
São Paulo (2006-2010)	–	–	–	–
Teresina (2002-2006)	77.0	23.1	84.2	15.8

- Data from Goiânia and São Paulo PBCR were not available

MV: Microscopic Verification; DCO: Death Certification Only
